# Hemodynamic Phenotypes in Congenital Diaphragmatic Hernia: Unresolved Questions and Future Directions

**DOI:** 10.3390/children13091137

**Published:** 2026-08-25

**Authors:** John T. Wren, Neil Patel, Patrick J. McNamara, Patrick Sloan

**Affiliations:** 1 Department of Pediatrics, University of Iowa, Iowa City, IA 52240, USA; 2 Department of Neonatology, Royal Hospital for Children, Glasgow G51 4TF, UK; 3 Department of Pediatrics, Washington University in St. Louis, St. Louis, MO 63110, USA

**Keywords:** congenital diaphragmatic hernia, hemodynamic phenotypes, echocardiography, pulmonary hypertension, neonatal hemodynamics

## Abstract

**Highlights:**

**What are the main findings?**

- Congenital diaphragmatic hernia is increasingly recognized as a dynamic cardiopulmonary disease in which lung function, pulmonary vascular disease, and cardiac dysfunction interact over time.
- Current hemodynamic phenotyping frameworks are clinically useful but remain limited by single-time-point static echocardiographic assessments that ignore evolving physiology.

**What are the implications of the main findings?**

- Phenotype-directed approaches may provide a framework for physiology-guided care within multidisciplinary CDH programs though this requires prospective validation.
- Future research should prioritize standardized echocardiographic and phenotype definitions, longitudinal multicenter assessment, impact on therapeutic decision-making, and mechanism-based approaches to precision cardiopulmonary care in CDH.

**Abstract:**

Congenital diaphragmatic hernia (CDH) is increasingly recognized as a dynamic cardiopulmonary disease in which pulmonary hypoplasia, pulmonary hypertension, and cardiac dysfunction interact to shape clinical instability, therapeutic response, and outcomes. Hemodynamic phenotyping has emerged as a strategy to move beyond binary classification of pulmonary hypertension and toward physiology-directed care. Early single-center experiences suggest potential clinical utility of echocardiography-guided, phenotype-directed management; however, external validation remains limited. Further important challenges remain, including technical and institutional barriers to timely echocardiography, limitations of static single-time-point assessments, uncertainty regarding what exactly defines each phenotype, and incomplete understanding of the impact of time and therapies on phenotype presentations. In this perspective, we summarize the evolution of heart-focused care in CDH, describe current hemodynamic phenotyping approaches, examine unresolved questions in phenotype classification and implementation, and outline future research priorities needed to advance dynamic, mechanism-based precision cardiopulmonary care for infants with CDH.

## 1. Introduction

Congenital diaphragmatic hernia (CDH) is a complex developmental disease process whose morbidity and mortality have historically been attributed primarily to the severity of pulmonary hypoplasia and pulmonary hypertension (PH) [1]. Reflecting this paradigm, advances in management have largely focused on lung-protective ventilation and strategies to reduce pulmonary vascular resistance (PVR) [2,3,4]. Although these approaches have contributed to improved survival, outcomes remain highly variable, with mortality still approaching 30% [5]. Beyond pulmonary hypoplasia alone, CDH is increasingly recognized as a dynamic cardiopulmonary disease in which pulmonary vascular disease, myocardial performance, and shunt physiology interact to shape response to therapies and clinical outcomes [2,6].

The past decade has seen a significant transition toward recognizing the major contribution of cardiac dysfunction to CDH pathophysiology. Animal studies, fetal and neonatal imaging, and large registry-based analyses have demonstrated that the fetal and neonatal heart in CDH faces unique developmental and physiologic challenges [7]. Abnormal in utero development contributes to myocardial hypoplasia and altered cellular structure [8], impaired left ventricular (LV) growth and function [9,10], as well as elevated right ventricular (RV) pressure owing to both intrinsically elevated PVR and redirection of fetal blood flow [11,12]. After delivery, LV hypoplasia as well as systolic and diastolic ventricular dysfunction are common [13,14,15] and are strongly associated with mortality [16]. Elevated PVR and pulmonary pressures further affect RV size and function, while ventricular–ventricular interactions may impair LV filling and performance [6,17]. As a result of these myriad effects, similar degrees of clinical and respiratory instability may arise from markedly different underlying hemodynamic states, each with distinct therapeutic implications.

This complexity has led to increasing interest in hemodynamic phenotyping as a strategy to classify infants with CDH into clinically meaningful physiologic subgroups [2]. Current phenotyping, however, remains largely dependent on static, single-point echocardiography-based assessments of a physiology that is dynamic and modified by temporal transitions and both pre- and post-echocardiogram clinical interventions.

In this perspective, we discuss the emergence of cardiac-focused care in neonates with CDH and address the major limitations of current phenotyping approaches including technical, physiologic, and implementation challenges. Where prospective evidence is lacking, the phenotypes and therapeutic implications proposed herein are discussed as hypothesis-generating concepts based on physiologic rationale, expert interpretation, and available observational data rather than validated clinical recommendations. We then examine the unresolved questions and future directions needed to bring precision cardiopulmonary care into the management of neonates with CDH.

### Literature Selection

A targeted literature review was performed via PubMed/MEDLINE through August, 2026 using combinations of terms related to “congenital diaphragmatic hernia,” “pulmonary hypertension,” “hemodynamics,” “echocardiography,” ventricular function, “cardiac dysfunction,” and “targeted neonatal echocardiography.” Additional studies were identified through review of reference lists from relevant articles and contemporary consensus statements. Priority was given to studies specifically evaluating neonatal CDH hemodynamics in the acute period, serial echocardiographic assessment, hemodynamic phenotypes and physiology-directed therapeutic strategies.

## 2. Toward Hemodynamic-Focused Care in CDH

The biologic nature of cardiorespiratory morbidity in infants with CDH is multi-factorial. High-fidelity care requires equal focus on pulmonary hypoplasia, pulmonary hypertension, and cardiac function. Gentle ventilation strategies with low mean airway pressure and maintenance of functional residual capacity (FRC) optimize heart–lung interactions and help to minimize PVR from under- or over-distention [18]. Heart-focused care in CDH emphasizes that inadequate gas exchange (hypoxemia, hypercarbia) is not only from pulmonary hypoplasia but also from inadequately supported hemodynamics. This includes a practice shift away from immediately adjusting ventilator settings with each abnormal blood gas and considering a more comprehensive assessment of cardiopulmonary status to guide the choice and timing of vasodilators, vasopressors, and inotropic medications to improve outcomes in infants with CDH.

While echocardiography has traditionally focused on binary identification and intervention for pulmonary hypertension, centers are increasingly incorporating cardiac function and shunt directionality into their care guidelines. This has impacted the timing and role of inhaled nitric oxide use [19,20], the initial choice of vasoactive medications [21], and the timing of surgical repair [22]. As seen in Table 1, Kuan and colleagues reported higher survival with echocardiography-guided care [23]; in addition, Sloan and colleagues recently reported higher survival and lower extracorporeal life support (ECLS) utilization following implementation of a treatment approach incorporating early echocardiography to guide inhaled nitric oxide (iNO) and inotropic support [24].

Echocardiography-based approaches to hemodynamic phenotyping have allowed clinicians to move beyond a binary assessment of pulmonary hypertension and toward a more integrated evaluation of cardiac performance, pulmonary vascular function, and shunt physiology to guide individualized management [6].

## 3. Hemodynamic Phenotypes in CDH

### 3.1. Three Canonical Phenotypes

**Figure 1:**
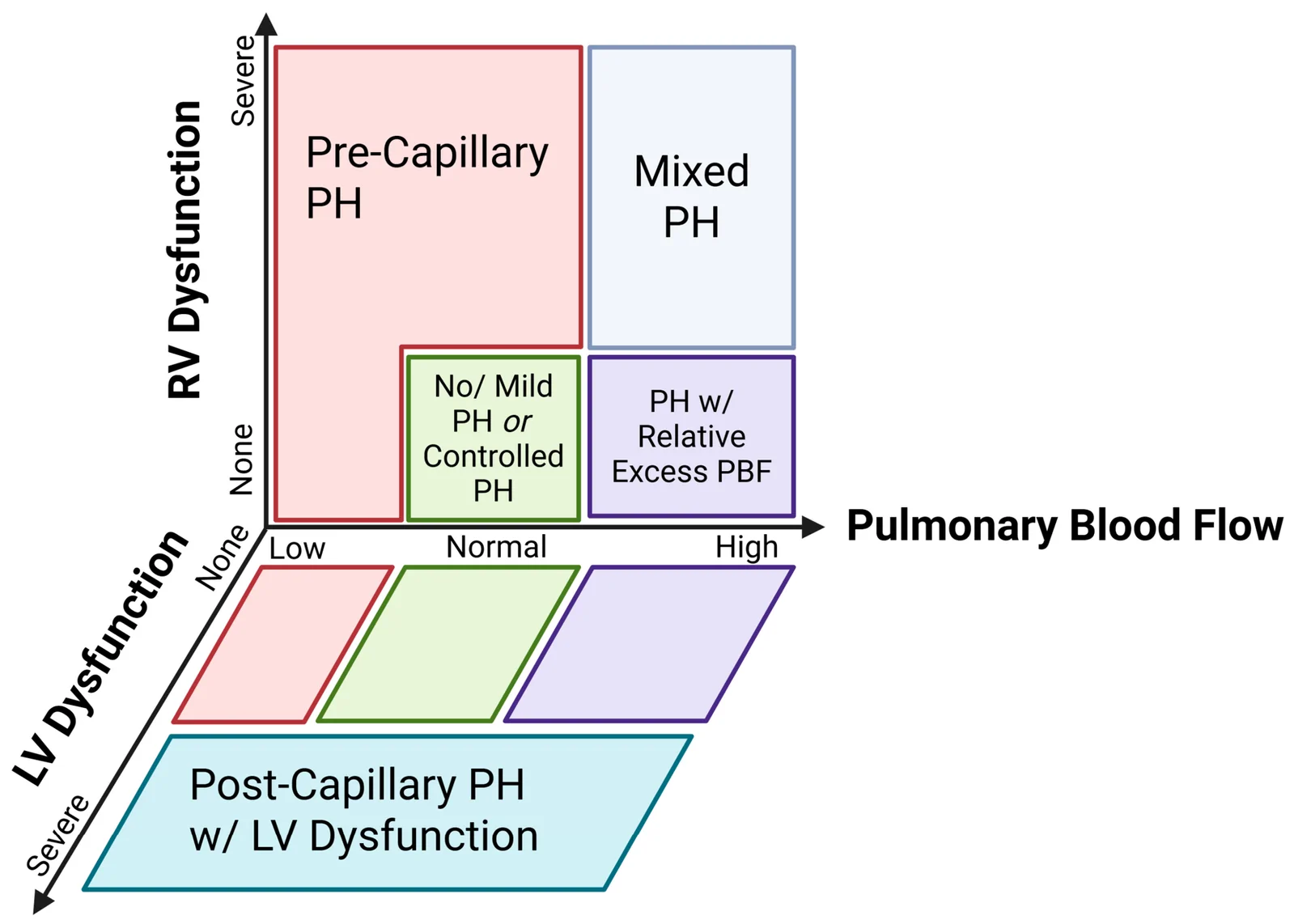
Proposed predominant hemodynamic phenotypes in congenital diaphragmatic hernia (CDH) in relation to biventricular dysfunction and degree of pulmonary blood flow. Depicted green, red, and blue are the three canonical phenotypes. Shown in gray and purple are proposed theoretical phenotypes that can develop over time in neonates with CDH. Overlap is likely between these physiologies and likely changes over time requiring the predominant underlying phenotype to be delineated at each time point.

Traditionally, using comprehensive echocardiography, three hemodynamic phenotypes in CDH have been described: (1) no/mild pulmonary hypertension (PH), (2) pre-capillary PH with or without RV dysfunction, and (3) post-capillary PH with LV dysfunction [23,27,28]. This first phenotype is characterized by absent or mild PH with preserved biventricular function and adequate pulmonary blood flow (PBF) (Figure 1). The pre-capillary PH phenotype is characterized by physiology consistent with predominantly elevated PVR, relatively impaired PBF, and frequently by RV dysfunction, which may be more likely to respond favorably to pulmonary vasodilators including iNO. Conversely, the post-capillary PH phenotype is characterized by physiology suggestive of elevated left-sided filling pressure in the setting of LV systolic dysfunction resulting in increased pulmonary venous pressure and a secondary elevation in pulmonary pressures. In this phenotype, pulmonary vasodilation may increase PBF and pulmonary venous return to a vulnerable LV, exacerbating left atrial (LA) hypertension and contributing to adverse outcomes. The traditional three-phenotype framework was developed as a pragmatic classification of the predominant hemodynamic physiology at the time of the initial echocardiographic assessment and was not presented with the potential to reflect dynamic changes over time.

### 3.2. Additional Proposed Phenotypes

**Figure 2:**
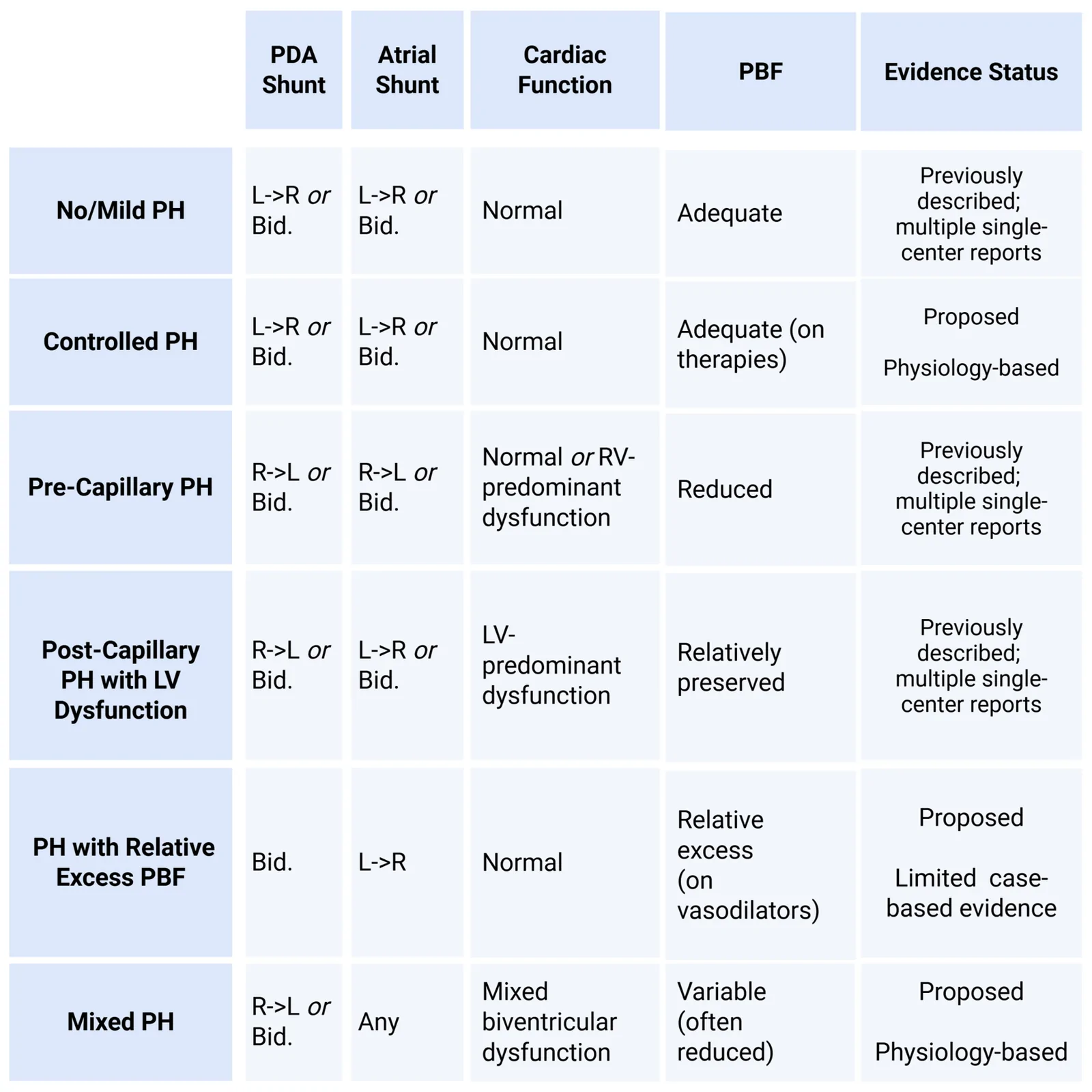
Proposed echocardiographic framework for adjudication of hemodynamic phenotypes in the acute care of neonates with CDH. These proposed features are intended as a conceptual framework for integrated echocardiographic phenotype adjudication and have not been prospectively validated as diagnostic criteria. No individual echocardiographic variable is sufficient to assign a phenotype, including shunt directions. Phenotypes are likely dynamic and can change over time. PH, pulmonary hypertension; PBF, pulmonary blood flow; L->R, left-to-right; Bid., bidirectional; R->L, right-to-left RV, right ventricle; LV, left ventricle; iNO, inhaled nitric oxide.

As seen in Figure 1, it is likely that this three-phenotype model is ultimately too simplistic to reflect the complexity of CDH hemodynamics. While not exhaustive, we propose additional phenotypes that require prospective validation. These include (1) controlled PH which we have included with the canonical no/mild PH phenotype in Figure 1 as it will appear similar on the echocardiogram (Figure 2). We define the controlled PH phenotype as neonates in whom treatment of elevated pulmonary artery pressure has led to preservation of ventricular function. In addition, we propose two additional phenotypes, namely (2) PH with normal LV function and relative excess PBF, and (3) pure mixed PH; these are shown in Figure 2 and described in detail later in the text. In the former, we propose that improvements in PVR post-natally coupled with the use of pulmonary vasodilators including iNO can lead in select patients to a relative excess of PBF returning to a vulnerable and often hypoplastic LV with intact systolic function resulting in elevated LV end diastolic and eventually pulmonary pressures. We define the latter mixed phenotype as one characterized by biventricular dysfunction in which a predominant underlying physiology cannot be readily ascertained from the echocardiogram at that timepoint.

Although these phenotypes are depicted in Figure 1 as discrete categories for clinical and graphical simplicity, CDH hemodynamics exist along a continuous and dynamic spectrum. Individual patients may demonstrate overlap between these categories, requiring integration of multiple findings to identify the predominant underlying physiology at a given time point. Importantly, predominant physiology may change throughout the clinical course in response to the postnatal transition, evolving cardiac function, medical therapies, and surgical repair. Accordingly, these proposed phenotypes should be interpreted as clinically useful descriptors of predominant physiology at a given time point rather than mutually exclusive or fixed biological entities. These proposed phenotypes represent a conceptual framework intended to generate testable hypotheses rather than validated diagnostic categories.

### 3.3. Phenotype Delineation

Hemodynamic phenotypes are adjudicated via a comprehensive echocardiographic assessment of ventricular function, cardiac output, estimations of pulmonary pressure, and shunt directions at the atrial and ductal levels [29]. Pulmonary blood flow is more challenging to calculate non-invasively via echocardiography but can be estimated by cardiac output assessment. In the absence of shunts, right and left ventricular output approximate pulmonary and systemic blood flow, respectively. As neonates with CDH will almost invariably have multiple early shunts [30], cardiac output interpretation must include shunt direction and magnitude of flow. With left-to-right ductal shunting, LVO becomes a more accurate measure of PBF while the converse is true for right-to-left PDA shunts where RVO more accurately estimates systemic blood flow. Each of these can further be altered by atrial shunt magnitude and direction. Accordingly, ventricular output measurements are interpreted together with ductal and atrial shunt direction and magnitude, chamber loading, and other supportive echocardiographic findings rather than direct measurements of PBF or systemic blood flow. Overall, there is a significant need for standardized phenotype definitions in CDH.

While echocardiography remains the primary method of hemodynamic assessment in neonates with CDH due to its widespread availability and non-invasive nature, important limitations must be recognized. Echocardiography can estimate pulmonary artery pressure using measures such as the tricuspid regurgitant jet, ductal shunt direction, and ventricular septal position; however, pulmonary pressure is not synonymous with PVR, PBF, or left-sided filling pressure. These latter variables are more commonly inferred from an integrated echocardiographic assessment of cardiac function, cardiac output, shunt directionality, chamber dimensions, and other supportive findings rather than measured directly. Definitive characterization of these hemodynamic variables requires invasive assessment via cardiac catheterization. Accordingly, the terms “pre-capillary” and “post-capillary” utilized in echocardiographic phenotyping should be interpreted as describing the predominant physiology inferred from echocardiographic parameters rather than a definitive invasive hemodynamic diagnosis. Within these limitations, post-capillary physiology is not assigned based on any single echocardiographic feature but rather requires a constellation of findings reflecting ventricular function, left-sided pressure-volume loading, and pulmonary pressure. Importantly, each of these are inherently load-dependent and thus the relative contribution of each must be interpreted within the broader clinical, physiologic, and echocardiographic context. Echocardiography thus provides a multidimensional physiologic assessment that can suggest, but cannot definitively distinguish, the relative contributions of PVR, PBF, and left-sided filling pressure.

### 3.4. Timing of Hemodynamic Reassessment and Phenotype Adjudication

The optimal timing and frequency of hemodynamic phenotyping in neonates with CDH have not been prospectively established. Given the temporal changes in cardiac function, loading conditions, PVR, and PBF that occur in neonates with CDH during the postnatal transition and in response to medical therapies, surgical repair, and other changes in loading conditions, it is physiologically plausible that hemodynamic phenotypes evolve over time. While not evidence-based, a pragmatic framework of echocardiography-guided hemodynamic assessment would include an initial assessment within the first 24 h, consistent with current consensus guidelines [31], and earlier assessment in the setting of significant clinical instability. The timing of subsequent reassessment should depend on clinical status, although reassessment within the following 24–48 h is reasonable to establish the early physiologic trajectory. Thereafter, the frequency of echocardiographic reassessment should be individualized according to disease severity, clinical evolution, and center-specific practices rather than at fixed, predetermined intervals. Additional assessments may be considered following major changes in clinical status or cardiopulmonary therapies, initiation of ECLS, and before and/or after surgical repair. At each assessment, the physiologic and clinical context should be incorporated into the echocardiographic interpretation to distinguish evolution of the underlying disease process from changes related to treatment exposure.

### 3.5. Ventricular Dysfunction in CDH

As one of the primary drivers of hemodynamic phenotyping in CDH, cardiac dysfunction is common with one study identifying biventricular dysfunction as the most common (19%), followed by RV dysfunction (15%), and isolated LV dysfunction (5%) in the first 48 postnatal hours [14]. The etiology of this dysfunction is multi-factorial. While there are shared features, including intrinsic alterations of the myocardium and myocardial capillary network [32], the etiology of dysfunction differs in part between the right and left ventricle.

RV dysfunction is likely primarily driven by elevated PVR resulting in increased afterload, to which the neonatal RV is uniquely vulnerable [33]. With increasing RV afterload, the RV may maladapt through myocardial hypertrophy, dilation, and tachycardia [34]. Once adaptation has been exceeded, RV dysfunction will follow. Beyond maladaptation, increased RV afterload will also narrow the differential between the RV/right atrium (RA) and aortic root end diastolic pressure, thereby decreasing coronary perfusion pressure to the right coronary artery, further impacting function [35]. Pulmonary hypertension in CDH persists following repair in the most severe patients and RV dysfunction may persist longer than the pure LV phenotype, until pulmonary pressures drop [30].

**Figure 3:**
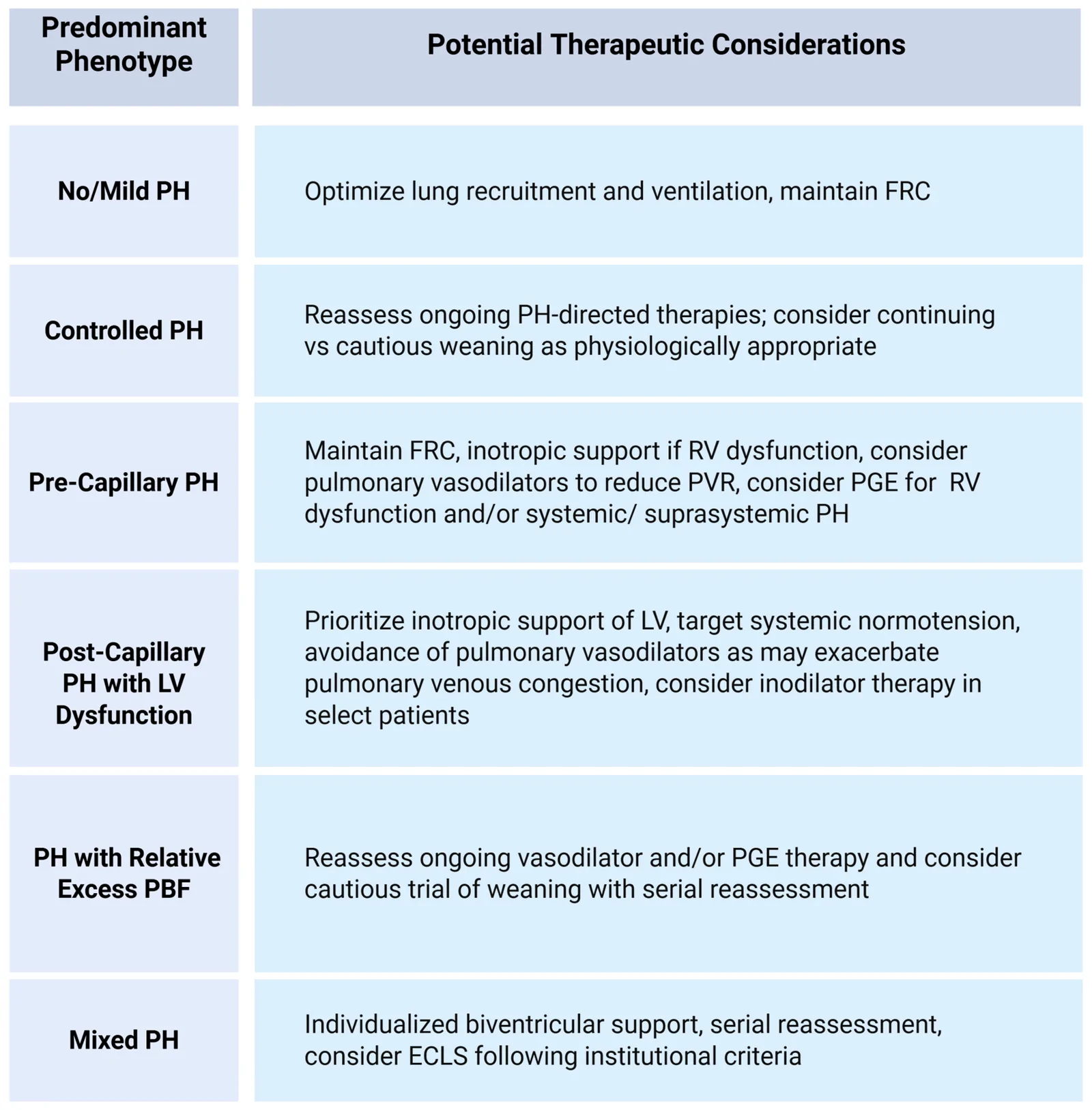
Proposed dynamic physiology-guided management framework for neonates with CDH. Therapeutic considerations are hypothesis-generating and reflect physiologic rationale, available observational evidence, and reported center-specific practices rather than prospectively validated treatment recommendations. Phenotype assignment reflects the predominant physiology at a given time point and should be reassessed following changes in clinical status, therapeutic interventions, or surgical repair. PH, pulmonary hypertension; FRC, functional residual capacity; PVR, pulmonary vascular resistance; PGE, prostaglandin E1; RV, right ventricle; LV, left ventricle; iNO, inhaled nitric oxide; PBF; pulmonary blood flow; ECLS, extracorporeal life support.

LV dysfunction appears to occur more acutely in the postnatal period, in contrast to RV dysfunction, likely as it is driven in part by LV hypoplasia [36] and abnormal LV myocardial structure [8] that is poorly adapted to the elevated systemic vascular resistance following cord clamping. Further, as the RV dilates it can bow into the LV further compromising LV filling and function via ventricular interdependence. This is coupled with decreased venous return to the left atrium when PVR fails to fall and pulmonary blood flow is slow to increase postnatally [37]. A vicious cycle develops wherein systemic perfusion is compromised resulting in acidosis and hypoxemia which further worsens LV function. Further, a recent study by Shalaby and colleagues found that while LV dimensions improved soon after surgical repair, there was also a transient impairment of LV function that requires further investigation [38]. This physiology raises concern that pulmonary vasodilation with iNO could increase pulmonary venous return to a vulnerable LV and provides a physiologic rationale for considering greater emphases on inotropic support [20,28]. A physiology-based, hypothesis-driven management approach is depicted in Figure 3. These reflect physiologic rationale, available observational evidence, and reported center-specific practices rather than prospectively validated treatment recommendations.

### 3.6. Significance of Patent Foramen Ovale (PFO) Shunt

As both the pre- and post-capillary PH phenotypes are associated with elevated pulmonary pressures and bidirectional (or even all right-to-left) patent ductus arteriosus (PDA) shunts, atrial shunt direction can provide key complementary information when interpreted within a broader echocardiographic assessment [2,29]. Interatrial flow is determined by a complex interplay of dynamic pressure and compliance relationships between the right and left heart, cardiac function, as well as cardiorespiratory interactions [39]. Physiology predominantly consistent with elevated PVR and relatively impaired PBF is associated with greater right-sided pressure and volume loading that favors predominant right-to-left atrial shunting. Conversely, LV dysfunction and physiology suggestive of elevated left-sided filling pressure will generally favor predominant left-to-right atrial shunting [2,28]. Importantly, atrial shunting in neonates with CDH is frequently bidirectional and thus this marker must be integrated within a comprehensive and multiparametric echocardiographic assessment to adjudicate predicted hemodynamic phenotypes. Finally, left-to-right shunting at the atrial level must be interpreted in context particularly in more mild patients with intact LV function [28].

### 3.7. Early Clinical Use

Clinically using these phenotypes to direct care has recently emerged with single-center reports suggesting potential clinical utility [20,23,27,28] (Table 1). Kuan and colleagues reported improved survival when serial echocardiographic hemodynamic assessments were introduced [23]. Lawrence and colleagues found that targeted use of iNO was associated with improved survival in a physiologically selected subgroup [20]. Sloan and colleagues similarly reported higher survival and lower ECLS utilization following implementation of an echocardiography-guided care strategy emphasizing early recognition and treatment of cardiac dysfunction [24] while Wren and colleagues found that use of iNO in infants with early post-capillary PH was associated with increased mortality [28]. Conversely, other single-center experiences have not demonstrated benefit from earlier or more frequent echocardiographic assessment. Yang and colleagues reported lower ECLS utilization and higher survival during an era in which the initial echocardiogram was delayed, although this change occurred alongside broader institutional practice changes [26]. Critically, these data are derived from retrospective, observational, single-center cohorts and are therefore subject to selection and reporting bias as well as observed associations that may be influenced by center-specific practice trends in medical and surgical management. In the absence of multicenter prospective studies, these studies collectively suggest the potential clinical value of phenotype-directed care while also highlighting the limited external validation of the proposed phenotype framework and therapeutic strategies.

## 4. Challenges of Hemodynamic Phenotyping in CDH

### 4.1. Institutional Requirements

Herein, we propose that the optimal care and outcomes for neonates with CDH includes delivery and management in a center with access and expertise in hemodynamic-directed care as part of an interdisciplinary CDH team. Important barriers remain, however, in the implementation and use of hemodynamic phenotype-directed care in CDH. There remains worry about the availability of echocardiography, agitation of a critically ill neonate, the interpretation of the assessment, and whether the interventions will actually improve care. To successfully implement heart-focused care at individual institutions, each of these barriers will need to be overcome. The increasing availability of hemodynamics programs and targeted neonatal echocardiography has improved clinician access to high-fidelity, multiparametric echocardiography that is then interpreted by an intensivist who understands the complete clinical picture. In the absence of this expertise, a partnership must be formed with Pediatric Cardiology to ensure availability of both the acquisition and interpretation of echo data, sufficient to differentiate phenotypes, in a timely manner.

As CDH is a relatively low-frequency event, high-volume centers increasingly utilize specialized multidisciplinary care teams for neonates with CDH to optimize outcomes [24,40,41]. At centers without neonatal hemodynamics programs, the challenge of incorporating the echocardiography report will fall upon these clinicians to integrate the findings into their comprehensive care for infants with CDH. As echocardiography alone will not improve outcomes, institutional and program focus should be directed toward echocardiography-based characterization of the underlying phenotype followed by targeted and timely intervention. A small and highly trained expert group with multidisciplinary collaboration is needed to bring heart-focused, phenotype-directed care and to provide precision medicine in this fragile population.

### 4.2. Technical Challenges

Phenotype identification is not possible by clinical assessment alone [28] and requires direct cardiac imaging with accurate interpretation. Echocardiography is the only feasible modality in the acute setting of early stabilization in a neonate with CDH but has technical and practical limitations. Even in the absence of structural congenital heart disease, pre-repair echocardiograms are confounded by distorted and displaced cardiac anatomy. Determination of atrial shunt direction, a critical component of CDH hemodynamic phenotyping [6], can be particularly challenging in this population. Although typically assessed from a subcostal view angled toward the left shoulder, it is often best visualized from the right axilla angled toward the left lateral chest wall in infants with left-sided CDH. These technical challenges are frequently compounded by an “after hours” need for echocardiography assessment when expert sonographers may not immediately be available. This has led to concerns regarding prolonged image acquisition time and potential impact on clinical stability such that select centers are delaying the initial and subsequent echocardiograms [26].

While critically important, echocardiography measurements in CDH are also inherently more challenging to perform and interpret. Ventricular function in current clinical practice is often defined either subjectively or via single metrics for the right and left ventricle, respectively. While these data may provide actionable information, it remains an incomplete assessment of cardiac function in infants with CDH; in addition, there is risk of subjective misinterpretation. Rather, quantitative and multiparametric measures of biventricular function are critical but must be interpreted in context. Standard techniques are key to optimize in CDH including proper Doppler alignment and sample gate positioning with tissue Doppler imaging in abnormally angulated CDH anatomy. Further, a metric such as tricuspid annular plane systolic excursion (TAPSE), a well-supported measure of RV systolic function [42], is angle-dependent and indexed to gestational age-specific RV length, both of which are likely to be abnormal in CDH. It is important to interpret such metrics alongside CDH-specific normative/comparative data where available [7].

### 4.3. How Can Hemodynamic Phenotype-Based Care Be Applied at Centers Without a Neonatal Hemodynamics/Targeted Neonatal Echocardiography (TNE) Program?

Despite these challenges, there is growing interest at many centers in incorporating hemodynamic-directed care in neonates with CDH. However, most level IV NICUs, particularly in the United States, do not have a formal hemodynamics/TNE program as these require formal competency-based training for neonatologists practicing TNE, including proficiency in image acquisition, interpretation, quantitative measurement, and integration of echocardiographic findings [42]. There remain too few neonatologists with this skill set to cover the current needs. At centers without established neonatal hemodynamics programs, partnership with existing Pediatric Cardiology infrastructure provides a practical pathway to implementing a hemodynamic phenotype-focused approach to infants with CDH.

Communicating with Pediatric Cardiology regarding the key information requested from a CDH echocardiogram is critical. A focused echocardiography protocol can be implemented that will allow identification of phenotypes and guide management [29]. The minimum information needed will require evaluation of atrial and ductal shunt directionality (ideally quantitative or at minimum including subjective predominant direction), ventricular septal position, objective measures of LV function, and ideally quantitative measures of RV function (Table 2). Interpretation of these measures can demonstrate intra-reader variability; therefore, partnership with a core cardiology reading group may help remove this variability. Regardless of the model used, implementation should incorporate standardized image acquisition and reporting protocols with ongoing quality assurance under the oversight of Pediatric Cardiology.

A guideline for care that emphasizes the identification of the different cardiac and pulmonary hypertension phenotypes can be implemented with Pediatric Cardiology partnership to read echocardiograms in real-time. This will allow identification and subsequent follow-up of response to interventions. Recognizing that the echocardiogram is merely a tool at a singular point in time, its power in affecting outcomes lies in the understanding and interpretation of this tool, as well as choosing the appropriate intervention.

In sum, broader implementation will require standardized echocardiographic acquisition and interpretation, appropriate training and quality assurance, and close collaboration between neonatal hemodynamics and Pediatric Cardiology programs.

## 5. What Remains Unresolved in CDH Hemodynamic Phenotyping?

Hemodynamic phenotyping has provided an important framework for moving beyond binary classification of pulmonary hypertension in CDH and toward physiology-directed care. However, several major questions limit its current clinical and investigative application. Addressing these questions is essential if CDH phenotyping is to progress from descriptive classification toward precision cardiopulmonary care.

### 5.1. Are Currently Proposed CDH Hemodynamic Phenotypes Biologically Distinct or Temporally Evolving States?

The current paradigm of CDH hemodynamic phenotypes is largely derived from a static, single-time-point echocardiographic assessment [2,27]. It is unclear whether currently proposed phenotypes represent distinct biological endotypes or rather dynamic physiologic states that evolve over time in response to intrinsic changes in myocardial function, PVR, and medical therapies.

Answering this question is challenging, in part because there remains limited consensus regarding the optimal timing of the initial echocardiogram and whether and/or when follow-up assessments should be performed [26,31]. Further, registry-based studies of CDH echocardiograms are limited by the restricted amount of information collected in these databases. Several recent single-center longitudinal studies of CDH echocardiograms have begun to answer this question. Sloan and colleagues found normalization of isolated LV dysfunction in all neonates and normalization of isolated RV dysfunction in all but one neonate within the first 72 h. Biventricular dysfunction more frequently persisted and was associated with a greater need for ECLS [24]. Le and colleagues demonstrated temporal improvements in biventricular systolic function with a median time to normalization of 2 and 1 days for the RV and LV, respectively; however, 60% of neonates continued to have at least systemic PH by day 3. This study was not designed to differentiate pre- versus post-capillary etiologies [30]. Toyoshima and colleagues similarly reported serial improvement in RV function but interestingly did not observe this for the LV. Instead, using more advanced three-dimensional echocardiography, they found improvements in LV volume metrics, suggesting LV growth or improved filling rather than an overt improvement in LV contractility [43]. Echoing this, Pugnaloni and colleagues found that increases in LV size were associated with reduced mortality and reduced need for extracorporeal life support (ECLS) [44].

To date, detailed studies of longitudinal hemodynamic phenotypes and natural history in CDH are largely lacking. Our group, as well as Ibarra-Rios and colleagues, have previously described the initial (<24 h) hemodynamic phenotype using comprehensive TNE [27,28]; however, this ignores the potential contributions of physiologic changes in loading conditions after stabilization, surgical repair, and the effect of medications—specifically, whether phenotypes remain static until normalizing or the transition between underlying pathophysiologies is unknown. Further study of longitudinal changes in hemodynamic phenotypes is needed.

### 5.2. How Exclusive Are Hemodynamic Phenotypes in CDH?

While these three hemodynamic phenotypes have begun to be adopted in clinical practice, phenotype adjudication can be challenging and complex. This is particularly relevant when relying on assessments and measurements that are not yet standardized or defined for CDH as well as shunt directionality that, in one study, was bidirectional in 95% and 45% of initial ductal and atrial shunts, respectively [30]. While not often discussed, a pure “mixed phenotype” must be considered, particularly in settings of profound biventricular dysfunction (Figure 1). Quantitative and multiparametric measures of ventricular function can help to identify the predominantly affected ventricle; however, this is not always possible. Indeed, these patients tend to have the highest risk of mortality [45] and center-specific practice suggests earlier utilization of ECLS though this approach has not been prospectively evaluated [22].

### 5.3. Can Our Therapies Induce Altered Hemodynamic Pathophysiology in CDH?

While iNO use is declining, a recent study found that treatment was still employed in 43% of neonates with CDH [46]. Prostaglandin (PGE) therapy is also used in some centers in this population for maintenance of ductal patency and optimization of pulmonary blood flow in patients with severe RV dysfunction and/or refractory PH, and optimization of systemic blood flow in the setting of moderate/severe LV dysfunction [47]. When used in physiologically targeted patients, iNO and PGE have been associated with improvements in selected clinical and hemodynamic outcomes in single-center reports, including markers of oxygenation and PH; however, evidence for an independent effect on survival remains limited [20,47,48]. Levinkopf and colleagues recently demonstrated that targeted PGE use for RV support with standardized initiation and discontinuation parameters (and a median duration of 7 days) was associated with improved hemodynamics without adverse outcomes [49]. The impact on the vulnerable CDH LV when PGE is used for LV support or in a less targeted/more prolonged manner is less investigated.

If ventricular function and PVR improve over time and after intervention, blood flow returning to the left ventricle increases progressively [43]. Even with intact systolic function, this can raise LV end diastolic volumes, leading to left atrial hypertension and upstream elevated pulmonary pressures. This is particularly relevant in the setting of a large PDA with a significant left-to-right shunt component (even if bidirectional) if vasodilator therapy is not weaned appropriately. Reflecting this, there are case reports of term neonates with CDH requiring PDA-directed pharmacotherapy and/or percutaneous device closure to mitigate pulmonary overcirculation [50,51].

As pulmonary pressures remain elevated, a shift in atrial shunt direction to left-to-right while remaining on vasodilators such as iNO and/or PGE becomes the key discerning factor suggesting that the underlying physiology has changed. This transition toward a more post-capillary etiology with relative excess PBF to a vulnerable LV even with normal systolic function (Figure 1) raises the hypothesis that ongoing vasodilator therapy may be maladaptive and that reassessment or weaning of vasodilator therapy should be considered. Further research is needed to determine whether vasodilator therapy-associated PH physiology represents a distinct phenotype, a transient treatment effect, or an expected stage in recovery from severe pre-capillary PH.

### 5.4. Is There a Contribution of Diastolic Dysfunction to CDH Hemodynamic Phenotypes?

As we have discussed above, heart-focused care in CDH has progressed over the past decade, tied tightly to a focus on left and right ventricular systolic function [6,29]. Diastolic dysfunction is increasingly recognized in other disease processes in neonatology, including bronchopulmonary dysplasia [52]; however, its contribution to CDH hemodynamics is under-investigated. Two studies from Cantone and colleagues as well as Tyden and colleagues recently identified abnormal markers of LV diastolic function in neonates with CDH compared to healthy controls in the first 24 and 72 h, respectively. Interestingly, an abnormal left atrial strain rate at 24–72 postnatal hours was shown to correlate with the need for invasive ventilation at 14 days and prolonged hospitalization [53,54].

While the post-capillary PH phenotype in CDH is typically based upon the premise of LV systolic dysfunction, leading to increased LV end diastolic pressure and volume, LV diastolic dysfunction with an elevated pulmonary capillary wedge pressure (PCWP) can cause the same result. This may be clinically important as LV diastolic dysfunction is much more subtle and challenging to identify echocardiographically. Both physiologies, however, have the potential to respond adversely to pulmonary vasodilator therapy leading to relative excess PBF and further exacerbation of LA hypertension and post-capillary PH. In a cohort of repaired neonates with CDH at a median of 78 days, Dias Maia and colleagues found diastolic dysfunction (e.g., elevated PCWP) in 53% who underwent cardiac catheterization [55]. Further study is needed, including evaluation of the RV.

### 5.5. Can a Hemodynamic Phenotype Be Identified from an Abridged Echocardiogram in an Unstable Infant with CDH?

The initial echocardiogram in a neonate with CDH can be time-consuming if not performed by a sonographer with expertise in the neonatal population. Indeed, the median duration of the initial echocardiogram was 52 min when performed by a Pediatric Cardiology trainee after hours in a recent study at our institution [56]. When performed by experienced sonographers, the median duration was 39 min while it reduced further to a median of 24 min when performed by a specially trained neonatologist via TNE. As TNE is not yet available at most centers, there is considerable interest in the minimum required images and measurements needed to adequately adjudicate a hemodynamic phenotype.

Depicted in Table 2 is a proposed focused set of images and measurements to assess biventricular function, pulmonary hemodynamics, shunt directions, and screen for congenital heart disease. At our center, we applied this approach as an initial feasibility assessment and observed an average duration of 7 min in four clinically unstable neonates with CDH [56]. This may offer a practical compromise between the need for early physiologic information and the risks of prolonged image acquisition in an unstable infant, though evaluation in a larger cohort is required.

This approach must be balanced against the significant risk of missing complex congenital heart disease, which is more common in neonates with CDH [57]. As such, any abbreviated echocardiogram should (1) only be utilized in an infant with a known fetal echocardiogram predicting structurally normal anatomy and (2) be followed by a complete echocardiogram once the infant is clinically stabilized.

## 6. Future Research Priorities

Advances in cardiorespiratory care for CDH have led to increasing interest in precision medicine and targeted hemodynamic-directed care for this high-risk patient population. Some early studies suggest potential benefit from physiology-guided management [20,23,24] while others suggest no benefit [26]. However, several critical gaps, summarized in Table 3, must be addressed before hemodynamic phenotyping can be routinely used to guide individualized therapy across centers.

A major priority is to define the natural history of hemodynamic phenotypes across the clinical course. Current studies are limited by inconsistent definitions and timing of echocardiography, heterogeneous clinical management, and reliance on single-time-point assessments. Multicenter longitudinal studies using standardized phenotype definitions, assessment time points, and clinical outcomes are needed to determine whether phenotypes represent stable biological endotypes or dynamic trajectories, and to externally validate proposed classifications in independent cohorts. Importantly, the physiologic and therapeutic context of each assessment should be documented to distinguish changes associated with the postnatal transition, treatment, surgical repair, or altered loading conditions from evolution of the underlying disease process and to determine whether early phenotypes predict longer-term cardiorespiratory outcomes.

A second priority is to determine whether phenotype-guided therapeutic strategies improve clinical outcomes. Single-center observational studies suggest potential clinical utility of phenotype-guided use of established therapies including iNO, PGE, and prostacyclins in neonates with CDH [20,47,48,49]. A standardized approach for defining phenotypes would provide a framework to prospectively evaluate whether phenotype-guided therapeutic strategies improve outcomes between centers. While novel therapeutics remain important [58], optimizing how currently available therapies can be targeted to the underlying physiology may offer a more immediate opportunity to advance care.

The relationship between fetal cardiovascular physiology and postnatal hemodynamic phenotypes also remains poorly understood, as prenatal risk assessment has largely focused on measures of lung size. Studies integrating fetal echocardiography with longitudinal postnatal phenotyping are needed to identify potential predictors of postnatal physiology and enable more proactive management.

Future studies should also evaluate whether phenotype-specific biomarkers can complement echocardiography. Serum biomarkers of myocardial stress, endothelial dysfunction, pulmonary vascular remodeling, and inflammation may help to identify biological endotypes, predict phenotype evolution, and monitor response to therapy [59].

Advances in artificial intelligence and quantitative image analysis may provide new avenues for hemodynamic phenotyping in neonates with CDH. Machine-learning approaches could integrate multiple echocardiographic, clinical, and fetal variables to identify hemodynamic phenotypes and potentially predict phenotype transitions and trajectories. Reflecting this possibility, Conte and colleagues recently applied machine learning methods to fetal and early postnatal variables to predict mortality and PH severity in neonates with CDH [60]. Additional technologies, including automated quantitative assessment of cardiac function, have potential to improve reproducibility and facilitate longitudinal assessment; however, these approaches remain investigational and will require validation in the neonatal and CDH population.

Finally, progress in CDH hemodynamic research will require dedicated, multicenter infrastructure. Existing registries have provided valuable insights but are limited by reliance on selected elements of echocardiography reports rather than direct image review. The development of a multicenter CDH TNE repository, ideally linked to standardized clinical data already collected, would substantially accelerate validation of phenotype definitions, assessment of interobserver reliability, and discovery of novel imaging markers. Such infrastructure would also allow future studies to evaluate whether phenotype-directed interventions truly improve outcomes beyond large-scale outcome association alone.

Together, as summarized in Table 3, these priorities have potential to move the field from descriptive hemodynamic classifications toward dynamic, mechanism-based precision medicine.

## 7. Conclusions: A Paradigm Shift in CDH Care?

Survival for infants with CDH has remained stagnant at 70–75% in large registry data [5]. To improve outcomes for the most severe cases, we must give equal attention to hemodynamic-focused care integrated with management of pulmonary hypertension and pulmonary hypoplasia. Echocardiography provides an opportunity to characterize this physiology, and individual centers have reported favorable outcomes following implementation of care strategies incorporating echocardiography to guide vasodilator, inotropic therapy, and repair timing, although current evidence remains observational and single-center [5,20,23,24,28,48,49].

Three priorities are particularly urgent to advance the field. First, standardized and reproducible definitions of CDH hemodynamic phenotypes must be developed via expert consensus and externally validated. Second, longitudinal studies are needed to define phenotype trajectories in the context of changes in underlying physiology, therapeutic exposures, and surgical repair status. Third, prospective studies should determine whether matching therapies to the predominant hemodynamic phenotype improves clinically meaningful outcomes. Addressing these priorities will be essential to determine whether hemodynamic phenotyping can evolve from a physiologic and hypothesis-generating framework into a validated approach to precision cardiopulmonary care in neonates with CDH.

## Figures and Tables

**Table 1:** Implementation and outcome measures for heart-focused care published by single-center cohorts.

	Study Design	Care Interventions	Role/Timing of Echocardiogram	Reported Outcomes
Wild et al. (2025) [25] *n* = 192	Retrospective, single center Three epochs	Golden-hour multidisciplinary care bundle Decreased empiric iNO use Initial FiO_2_ to 50%	Echo in first 24 h and prior to iNO initiation	No change in survival or ECLS utilization Decreased iNO use and initial FiO_2_
Kuan et al. (2024) [23] *n* = 20	Retrospective, single center Two epochs	Hemodynamic assessment Multidisciplinary rounds Jet ventilation for rescue	Targeted neonatal echo First 24 h Daily follow-up	Increased number of echocardiograms Improved survival (100%)
Yang et al. (2025) [26] *n* = 306	Retrospective, single center Two epochs	Delayed initial echocardiogram Part of broader CDH practice change	Initial echo delayed to a median of 40 h	Reduced ECLS utilization Improved survival Decreased use of iNO
Byrd et al. (2025) [19] *n* = 13	Retrospective, single center Two epochs	Low MAP vent strategy Limit iNO with LV dysfunction Inotrope-focused early care	Not specified	Decreased ECLS need Decreased iNO utilization Trend in survival (92%)
Sloan et al. (2026) [24] *n* = 53	Retrospective, single center Two epochs	Low MAP vent strategy Limit iNO with LV dysfunction Inotrope-focused care with cardiac dysfunction Serial echo	Cardiology read echo First 2 postnatal hours Daily follow-up	Decreased iNO use Decreased ECLS Improved survival (89%)

PH, pulmonary hypertension; iNO, inhaled nitric oxide; FiO_2_, fraction of inspired oxygen; ECLS, extracorporeal life support; LV, left ventricular; Echo, echocardiogram.

**Table 2:** Key echocardiographic images for hemodynamic phenotype adjudication in neonates with congenital diaphragmatic hernia.

Echo View	Image	Modality	Key Measurement	Clinical Interpretation
Apical	4-chamber	2D	EF by Simpson’s biplane ^1^	LV systolic function
			LV and RV dimensions	Cardiac chamber size
			RV FAC	RV systolic function
		M-Mode	TAPSE	RV systolic function
		Color and continuous-wave Doppler over TV	Peak TR velocity, estimated RVSP	Estimate of PA systolic pressure
		TDI	LV and RV free-wall s’	LV and RV systolic function
	5-chamber	Pulsed wave Doppler of LVOT	LVOT VTI	LV cardiac output
	LV-focused 2-chamber	2D	EF by Simpson’s biplane ^1^	LV systolic function
	RV-focused 3-chamber	2D	RV FAC	RV systolic function
		Color and continuous-wave Doppler over TV	Peak TR velocity, estimated RVSP	Estimate of PA systolic pressure
PLAX	LVOT	2D	Aortic annulus for cardiac output	LV cardiac output
	RVOT	Pulsed wave Doppler of RVOT	RVOT VTI	RV cardiac output
		2D	Pulmonic annulus for cardiac output	RV cardiac output
	Tricuspid valve	Color and continuous-wave Doppler	Peak TR velocity, estimated RVSP	Estimate of PA systolic pressure
PSAX	Papillary muscle level	2D	Septal position (sEI)	Estimate of pulmonary pressure relative to systemic
			RV dilation	Estimate of RV dilation relative to LV
	Tricuspid valve	Color and continuous-wave Doppler	Peak TR velocity, estimated RVSP	Estimate of PA systolic pressure
Suprasternal	Arch Sweep	2D, color Doppler, continuous wave Doppler	Aortic narrowing and gradient	Screen for arch abnormalities
	PDA	2D	Size	Estimate PDA size
		Pulsed wave Doppler	Spectral Doppler flow pattern	PDA shunt direction
	Crab view	2D + color Doppler	4 pulmonary veins draining to LA	Confirm anatomy
Subcostal	Atrial shunt	2D	Size	Estimate atrial shunt size
		Color	Shunt direction by color	Atrial shunt direction
	Sweep	Color	Confirm anatomy	

Depicted are key images and measurements needed for a quantitative and multiparametric assessment of ventricular function, pulmonary pressures, and shunt directions. Background highlighted metrics denote minimum required assessments for a rapid bedside phenotype interpretation. A complete echocardiogram should be performed once the neonate is stabilized. ^1^ Both the apical 4- and 2-chamber view are required to calculate left ventricular (LV) ejection fraction (EF) by Simpson’s biplane. TV, tricuspid valve; RV, right ventricle; FAC, fractional area change; TAPSE, tricuspid annular plane systolic excursion; TR, tricuspid regurgitant; PA, pulmonary artery; MR, mitral regurgitation; LA, left atrium; TDI, tissue Doppler imaging; PLAX, parasternal long axis; LVOT, left ventricular outflow tract; RVOT, right ventricular outflow tract; VTI, velocity time integral; PSAX, parasternal short axis; sEI, systolic eccentricity index; PDA, patent ductus arteriosus.

**Table 3:** Future directions and research priorities in CDH hemodynamic phenotyping.

Domain	Research Priority	Why It Matters
Definitions/Consensus	Standardize phenotype classifications	Enable multicenter comparisons
	External validation of proposed phenotypes	Establish reproducibility and generalizability across centers
	Natural history of phenotypes	Determine whether phenotypes are static or dynamic and whether changes represent disease evolution or changes in therapies and repair status
	Cardiac catheterization correlation with echocardiogram phenotypes	Enables gold standard validation of proposed phenotypes
	Proposed vasodilator therapy-associated post-capillary PH	Validation of potential clinically relevant novel phenotype
Mechanisms	Linking phenotype identification to therapeutic decision-making	Assess effectiveness of targeted therapies
	Fetal pathophysiology impact on postnatal presentation	Enable proactive care
	Diastolic dysfunction in phenotypes	Stratified post-capillary PH etiology
	Phenotype-specific biomarkers	Enable earlier identification and transitions
	AI-assisted phenotyping	Improves reproducibility, integrates multidimensional data
Infrastructure	Multicenter CDH-specific echocardiogram repository	Allows validation, reproducibility testing, and discovery

## Data Availability

No new data were created or analyzed in this study. Data sharing is not applicable to this article.

## Abbreviations

The following abbreviations are used in this manuscript:

CDH	Congenital Diaphragmatic Hernia
PH	Pulmonary hypertension
PVR	Pulmonary vascular resistance
LV	Left ventricle
RV	Right ventricle
FRC	Functional residual capacity
iNO	Inhaled nitric oxide
ECLS	Extracorporeal life support
PBF	Pulmonary blood flow
LA	Left atrial
TAPSE	Tricuspid annular plane systolic excursion
TNE	Targeted neonatal echocardiography
PGE	Prostaglandin E1
PDA	Patent ductus arteriosus
PCWP	Pulmonary capillary wedge pressure
